# GlycA Levels during the Earliest Stages of Rheumatoid Arthritis: Potential Use as a Biomarker of Subclinical Cardiovascular Disease

**DOI:** 10.3390/jcm9082472

**Published:** 2020-08-01

**Authors:** Javier Rodríguez-Carrio, Mercedes Alperi-López, Patricia López, Ángel I. Pérez-Álvarez, Miriam Gil-Serret, Núria Amigó, Catalina Ulloa, Lorena Benavente, Francisco J. Ballina-García, Ana Suárez

**Affiliations:** 1 Area of Immunology, Department of Functional Biology, Faculty of Medicine, University of Oviedo, 33006 Oviedo, Spain; lopezpatricia@uniovi.es (P.L.); anasua@uniovi.es (A.S.); 2 Bone and Mineral Research Unit, Instituto Reina Sofía de Investigación Nefrológica, REDinREN del ISCIII, Hospital Universitario Central de Asturias, 33011 Oviedo, Spain; catalinaulloac@hotmail.com; 3 Instituto de Investigación Sanitaria del Principado de Asturias (ISPA), 33011 Oviedo, Spain; mercedes_alperi@hotmail.com (M.A.-L.); jballina@telefonica.net (F.J.B.-G.); 4 Department of Rheumatology, Hospital Universitario Central de Asturias, 33011 Oviedo, Spain; 5 Department of Neurology, Hospital Universitario Central de Asturias, 33011 Oviedo, Spain; angelperez@telecable.es (Á.I.P.-Á.); lbf.benfer@gmail.com (L.B.); 6 Biosfer Teslab, SL, 43201 Reus, Spain; mgil@biosferteslab.com (M.G.-S.); namigo@biosferteslab.com (N.A.); 7 Metabolomics Platform, Universidad Rovira i Virgili (URV), Instituto de Investigación Sanitaria Pere Virigili (IISPV), CIBERDEM, 43007 Tarragona, Spain

**Keywords:** glycoproteins, early rheumatoid arthritis, cardiovascular risk, atherosclerosis, inflammation, H-NMR

## Abstract

This study aimed at evaluating the clinical relevance of glycoprotein profiles during the earliest phases of rheumatoid arthritis (RA) as biomarkers of cardiovascular (CV) risk and treatment response. Then, GlycA and GlycB serum levels were measured using 1H-nuclear magnetic resonance in 82 early RA patients, 14 clinically-suspect arthralgia (CSA), and 28 controls. Serum glycosyltransferase activity was assessed by a colorimetric assay. Subclinical CV disease was assessed by Doppler-ultrasound. We found that GlycA and GlycB serum levels were increased in RA (both *p* < 0.001), but not in CSA, independently of cardiometabolic risk factors. Increased serum glycosyltransferase activity paralleled GlycA (r = 0.405, *p* < 0.001) and GlycB levels (r = 0.327, *p* = 0.005) in RA. GlycA, but not GlycB, was associated with atherosclerosis occurrence (*p* = 0.012) and severity (*p* = 0.001). Adding GlycA to the mSCORE improved the identification of patients with atherosclerosis over mSCORE alone, increasing sensitivity (29.7 vs. 68.0%) and accuracy (55.8 vs. 76.6%) and allowing reclassification into more appropriate risk categories. GlycA-reclassification identified patients with impaired lipoprotein metabolism. Finally, baseline GlycA levels predicted poor clinical response upon anti-rheumatic treatment at 6 and 12 months in univariate and multivariate analysis. In sum, increased GlycA levels during the earliest stage of RA can be considered a powerful biomarker for CV risk stratification and treatment response.

## 1. Introduction

Rheumatoid arthritis (RA) is a chronic inflammatory condition. Protein post-translational modifications (PTMs) are a common hallmark of RA [1]. Although the role of protein citrullination is firmly established, less is known about other PTMs, such as glycosylation. Then, characterizing the human glycoproteome has emerged as a relevant source potential of biomarkers and disease mediators [2]. Recent evidence has found that protein glycosylation is involved in key biological processes such as cell adhesion and migration and signal transduction [3,4]. Glycosylation has also a role in controlling the immune response, and inflammation leads to extensive glycosylation changes on inflammatory proteins by glycosyltransferases [5]. The use of ^1^H-nuclear magnetic resonance (H-NMR) has allowed the identification of a prominent signal arising from the highly mobile acetyl groups of the N-acetylglucosamine and N-acetylgalactosamine, called “GlycA” [6]. This signal can be considered a broad surrogate marker of inflammation. However, its full clinical implications are to be characterized.

Different authors have already demonstrated higher levels of GlycA in rheumatic conditions [7,8,9,10], mostly conducted in patients with long-lasting conditions. This represents a major limitation to understanding the nature and clinical relevance of GlycA as a biomarker, in part owing to the broad exposure to treatments [11] and overt comorbidities. Moreover, in the setting of RA, notable differences were observed between established disease and the early stage, in terms of clinical phenotype and management. Interestingly, the early stage of the disease is of paramount importance for the long-term disease outcomes [12], and it has been found to be a significant predictor of sustained remission and better disease control. Therefore, characterizing biomarkers during early disease is a scientific priority.

GlycA analysis has shown promising results in the setting of cardiovascular (CV) risk [13]. CV risk stratification is suboptimal in RA, as current clinical risk scores do not account for the effect of inflammation and clinical features [14]. Imaging screening for asymptomatic atherosclerosis has been proposed to identify patients at risk. Among them, the assessment of carotid intima-media thickness (cIMT) by ultrasonography has become an affordable technique to evaluate atherosclerosis burden, in rheumatic and non-rheumatic populations, and provide additional value over existing algorithms. However, in a recent taskforce on CV risk management, a low level of agreement on the use of cIMT has been reached [15]. Moreover, serious limitations pertaining to its general use, reproducibility, and access exist. Then, it may be speculated that GlycA may facilitate CV risk identification in RA. However, no studies have been conducted to address this question in RA.

Taking all these ideas into account, we hypothesize that investigating the glycoprotein signals obtained by NMR during the early stage of RA will shed new light onto their potential use as a biomarker. In this setting, the identification of the clinically suspect arthralgia (CSA) individuals will help to delineate the timeframe of glycoprotein emergence during the earliest stages of RA as well as their clinical relevance, with a focus on CV risk and treatment response. Therefore, the aims of this study are to evaluate glycoprotein levels by H-NMR as well as their role as biomarkers of subclinical CV disease compared with the existing algorithms and treatment outcomes during the earliest phases of RA.

## 2. Experimental Section

### 2.1. Study Participants

Our study involved 82 early RA patients fulfilling the 2010 American College of Rheumatology (ACR)/European League Against Rheumatism (EULAR) RA classification criteria [16] recruited at disease onset from the early arthritic clinic of the Department of Rheumatology at Hospital Universitario Central de Asturias (HUCA). A complete clinical examination, including Disease Activity Score 28-joints (DAS28), Simplified Disease Activity Index (SDAI), and Health Assessment Questionnaire (HAQ) calculations, was performed on all patients during the clinical appointment. Patients who were not exposed to disease-modifying antirheumatic drugs (DMARDs) and glucocorticoids at recruitment (treatment-naïve early RA patients) were prospectively followed up for one year upon conventional synthetic DMARD (csDMARD) therapy. Clinical outcomes, including DAS28, SDAI, and EULAR response criteria, were registered at 6 and 12 months. Clinical management was performed according to EULAR recommendations [17]. Patients achieving a DAS28 < 2.6 were considered as reaching remission. Subjects with CSA (*n* = 14) were recruited from the same clinic if they met at least four criteria of the EULAR definition of arthralgia suspicious for progression to RA [18]. Healthy controls (HCs) (23 women/5 men, mean age 53.30 ± 8.79 years) were recruited among age- and sex-matched healthy individuals from the same population.

A fasting blood sample was collected from all individuals by venepuncture, and serum samples were immediately transferred to the laboratory and processed within less than 2 h. Serum samples were stored at −80 °C until experimental procedures. A conventional blood biochemical and lipid analysis was performed in all individuals. The presence of traditional CV risk factors (dyslipemia, hypertension, obesity, diabetes, and smoking history) based on national guidelines was obtained from the medical records. CV risk was assessed using the mSCORE [15] and patients were classified in risk categories according to the European Society of Cardiology (ESC) consensus [19].

The study was approved by the local institutional review board (Comité de Ética de Investigación Clínica del Principado de Asturias, ref PI16/00113) in compliance with the Declaration of Helsinki. All study subjects gave written informed consent.

### 2.2. Carotid Ultrasound Imaging

Doppler ultrasound assessment was performed in the sonography laboratory (HUCA) by an experienced user blinded to the status of the study participants. All measures were carried out in B-mode, online by the same operator using a Toshiba Aplio XG machine (Toshiba American Medical Systems, Tustin, CA, USA) with software version 5.1, equipped with a 7.5 Mhz linear probe. The right and left carotid arteries were scanned (transverse and longitudinal approaches) by the sonographer to evaluate the carotid intima-media wall thickness (cIMT) and plaque presence. The cIMT was bilaterally measured across 1–2 cm segments of the near and far walls of the distal common carotid artery and the far wall of the carotid bulb and the internal carotid artery on both the right and left sides, according to the “Mannheim Carotid Intima-Media Thickness Consensus (2004–2006)” [20], and the mean value was used. The reproducibility of the cIMT measurement was 89.0%. Atherosclerotic plaque was defined as a distinct area protruding into the vessel lumen at least 0.5 mm, with 50% greater thickness than the cIMT found in surrounding areas or the presence of a cIMT > 1.5 mm [20]. Total number of plaques and echogenic characteristics were registered. Plaque prevalence was considered as the frequency of patients exhibiting at least one plaque according to the previous consensus definition. Subjects with plaque and/or cIMT > 0.90 mm were considered as having subclinical CV disease. Plaque vulnerability was assessed by the ultrasound appearance of the plaques and these were classified as “low” or “high” risk [21].

### 2.3. Glycoprotein Profiling

Glycoprotein signals (GlycA and GlycB) were assessed as previously reported [22]. In brief, serum samples were first diluted with deuterated water and 50 mM pH 7.4 phosphate buffer solution before H-NMR analysis. Then, H-NMR spectra were recorded at 310 K on a Bruker Advance III 600 spectrometer operating at a proton frequency of 600.20 MHz according to previously optimized experimental parameters for one-dimensional H-NMR pulse experiments. We analysed the region of the 1H-NMR spectrum where the glycoproteins resonate (between 2.15 and 1.90 ppm of the chemical shift [23]) using several analytical functions. For each function, we determined the total area (proportional to concentration), height, position, and bandwidth. The area of the GlycA arose from the number of protein–sugar bonds from the acetyl groups of N-acetylglucosamine and N-acetylgalactosamine, and the area of GlycB arose from those of N-acetylneuraminic acid. GlycA and GlycB NMR-derived areas were transformed to protein–sugar bond absolute concentrations (mol/L) using internal calibration. Height to width (H/W) ratios were also reported, being a parameter associated with the aggregation state of the sugar–protein bonds (higher H/W ratios indicate higher sugar–protein bond mobility). Height was calculated as the difference from baseline to the maximum of the corresponding NMR peaks and the width value corresponds to the peak width at half height.

### 2.4. Lipoprotein Characterization

An advanced lipoprotein characterization by means of the NMR-based Liposcale test [24] was performed. This protocol allowed the assessment of lipid content (cholesterol and triglycerides) of very low density lipoproteins (VLDL), intermediate density lipoproteins (IDL), low density lipoproteins (LDL), and high density lipoproteins (HDL), as well as the particle number and size (diameter) of VLDL, LDL, and HDL and their subclasses (small, medium, and large). To this end, serum samples were diluted as commented for glycoprotein analyses and processed as previously reported [24].

### 2.5. Serum Glycosyltransferase Activity

The glycosyltransferase enzymatic activity was quantified in serum samples by means of a colorimetric assay based on [25] using a commercial kit (Glycosyltransferase Activity Kit, reference EA001) from R&D systems (Wiesbaden, Germany).

The enzyme activity of glycosyltransferases in serum samples (diluted 1:4) was assayed using 10 mM uridine 5′-diphospho galactose (Sigma Aldrich, Overijse, Belgium) as donor substrate and 4 mM 4-Nitrophenyl-N-acetyl-b-d-glucosaminide (Sigma) as acceptor [25] for 30 min at 37 °C. Then, a specific coupling phosphatase was used to remove inorganic phosphate from the leaving nucleotide diphosphate (UDP). Next, the released inorganic phosphate was detected by the Malachite Green phosphate detecting reagents, which produce a colorimetric signal at 620 nm. A standard curve with known concentrations of inorganic phosphate (100–1.56 M) was assayed in parallel. The amount of inorganic phosphate released by the phosphatase equals the nucleotide sugar consumed during the first step, so the rate of inorganic phosphate production reflects the kinetics of that of the glycosyltransferase reaction. To compensate for the potential effect of serum phosphatases and background signal, each sample was analysed twice, with and without the coupling phosphatase, and the difference was used. The glycosyltransferase activity was expressed as phosphate concentration equivalents produced per minute (M P/min). Intra- and inter-assay reproducibility were <12% and <15%, respectively.

### 2.6. Statistical Analyses

Variables were tested for normality by means of the Kolmogorov–Smirnov test (with Lilliefors correction) prior to analyses. Variables following a normal distribution were expressed as mean ± standard deviation and analysed by parametric methods (one-way analysis of variance (ANOVA) tests), whereas those not following normality were summarized as median (interquartile range) and non-parametric tests were used (Mann–Withney U or Kruskal Wallis tests). Categorical variables were summarized as *n* (%) and analyzed by means of 2 tests. Correlations were assessed by Spearman ranks tests. Correlograms and network analyses were built to analyze the correlations among genes, as well as to visualize the associations among them in the different conditions. In order to add the GlycA levels to the mSCORE algorithm, GlycA categories were defined from the distribution of the GlycA levels observed in the HC population and a score was given as follows: Q1 (<618.83 mol/L): −1, Q2 (618.83–734.55 mol/L): +0, Q3 (734.55–822.36 mol/L): +1, and Q4 (>822.36 mol/L): +2. GlycA scores were added to the mSCORE values (mSCORE + GlycA).

The associations between glycoprotein levels and subclinical CV disease or treatment response were analysed by multiple logistic regression, and adjusted odds ratios (ORs) and 95% confidence intervals (CIs) were calculated. Confounders were entered as covariates in the logistic regression, so final models were fully adjusted. The discrimination ability for subclinical CV disease was assessed using the area under the receiver operating characteristic curve (AUC ROC). The performance of the classification of mSCORE and mSCORE + GlycA was analysed by classification measures (sensitivity, specificity, % patients correctly classified, and likelihood ratios), Matthews correlation coefficient, goodness of fit (Hosmer–Lemeshow test), and the Youden index to determine the optimal cut-offs (threshold points with maximum accuracy). Net reclassification improvement (NRI) and integrated discrimination improvement (IDI) with corresponding bootstrap 95% CI were computed from the predicted probabilities retrieved by logistic regression analyses. NRI quantifies the correctness of upward and downward movement of the predicted probabilities, whereas IDI assesses the size of changes in these probabilities. *p*-Value < 0.050 was considered as statistically significant. Statistical analyses were carried out under SPSS v. 23 (IBM, Armonk, NY, USA) and R v.3.6.3 (https://cran.r-project.org/).

## 3. Results

### 3.1. GlycA and GlycB Are Increased in Early RA Patients

**Figure 1:**
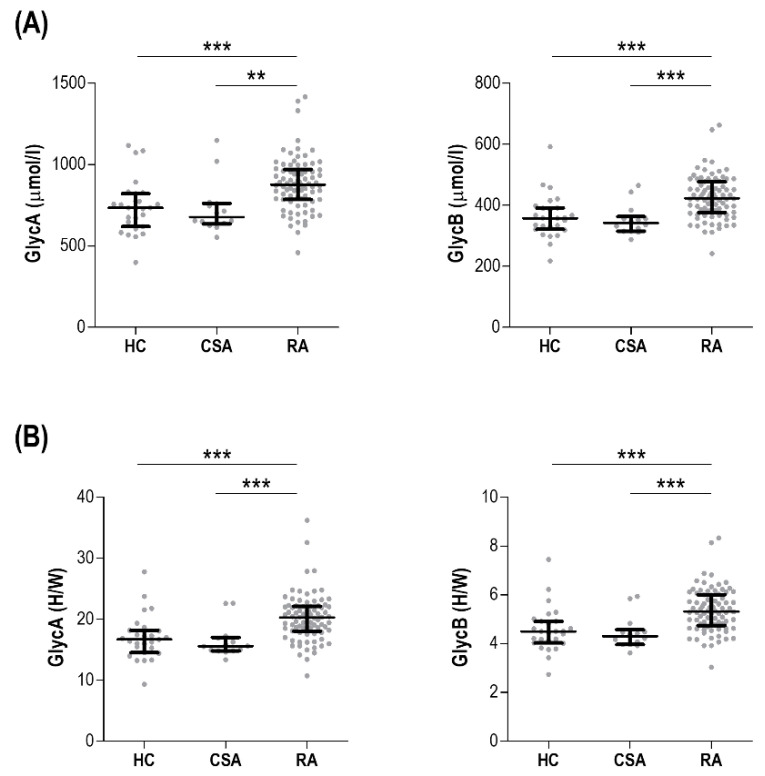
Comparative analyses of glycoprotein signals across study groups. Glycoprotein levels (GlycA and GlycB) measured as absolute levels (**A**) or height to width (H/W) ratios (**B**) were compared across healthy control (HC), clinically-suspect arthralgia (CSA) individuals, and early rheumatoid arthritis (RA) patients. Each dot depicts an individual, bars represent medians, and whiskers correspond to 25th and 75th percentiles. Differences were assessed by Kruskal–Wallis tests with Dunn–Bonferroni posthoc tests. The *p*-values from the latter were indicated as follows: ** *p* < 0.010 and *** *p* < 0.001.

The serum levels of glycoprotein signals were assessed in 82 early RA patients, 14 CSA individuals, and 28 HCs (Table 1). Both GlycA and GlycB absolute levels were increased in RA, but not in CSA (Figure 1A). Interestingly, the analysis of the peak shape ratios retrieved equivalent findings (Figure 1B). Similar results were obtained after excluding patients under csDMARD treatment (*n* = 13). On the other hand, no differences in the lipoprotein characterization were observed among groups (Table S1).

In RA patients, GlycA and GlycB levels were correlated with DAS28 (r = 0.306, *p* = 0.006 and r = 0.376, *p* < 0.001, respectively) and SDAI (r = 0.221, *p* = 0.032 and r = 0.279, *p* = 0.012, respectively). Similar associations were observed with erythrocyte sedimentation rate (ESR) (r = 0.331, *p* = 0.004, and r = 0.440, *p* < 0.001, respectively) and C-reactive protein (CRP) (r = 0.480, *p* < 0.001 and r = 0.531, *p* < 0.001, respectively). Interestingly, HAQ was observed to be correlated with GlycB (r = 0.239, *p* = 0.033), but not GlycA (r = 0.179, *p* = 0.094). No associations were retrieved for joint counts, pain assessment, or morning stiffness (all *p* > 0.050). There were no differences in glycoprotein signals between those recruited during the very early stage (<3 months since first symptoms reported) and the rest of patients (GlycA: 881.21(240.01) vs. 882.26(160.04) mol/L, *p* = 0.272 and GlycB: 424.68(55.51) vs. 415.91(123.70) mol/L, *p* = 0.332).

Furthermore, GlycA was correlated with ESR (r = 0.636, *p* = 0.014) and GlycB exhibited a correlation with CRP (r = 0.553, *p* = 0.040) in CSA subjects.

All these results support that GlycA and GlycB levels were increased in early RA, with strong correlations with disease activity and inflammation being retrieved, whereas no associations were found in CSA individuals.

### 3.2. GlycA, Traditional CV Risk Factors, and Glucose Homeostasis in Early RA

In order to evaluate whether the increased GlycA and GlycB levels in RA could be attributed to a higher burden of cardiometabolic risk factors, the associations between glycoproteins and traditional CV risk factors and glucose homeostasis parameters were evaluated.

No associations between traditional CV risk factors and glycoproteins levels were found in early RA patients (Table 2). GlycA was not correlated with fasting glucose levels, although a marginal association was observed for GlycB (Table 2). No associations were observed with insulin or C-peptide levels. Moreover, neither GlycA nor GlycB were correlated with glucose homeostasis indices, like the Homeostatic Model Assessment of Insulin Resistance (HOMA-IR) and the Quantitative Insulin Sensitivity Check Index (QUICKI) (Table 2).

### 3.3. GlycA and Subclinical CV Disease in Early RA

The associations between glycoprotein signals and subclinical CV disease in early RA patients were analyzed.

GlycA levels were positively correlated with cIMT (r = 0.312, *p* = 0.022) and higher levels were found in patients with plaque compared with their plaque-free counterparts (912.89(216.83) vs. 849.91(156.17) mol/L, *p* = 0.012). GlycA levels paralleled plaque number (r = 0.348, *p* = 0.002). As a consequence, RA patients with subclinical CV disease (plaque presence or cIMT > 0.90 mm) exhibited higher GlycA levels (921(210.20) vs. 846.74(133.67) mol/L, *p* = 0.018). Furthermore, patients with high-risk plaques exhibited higher GlycA levels than those with low-risk plaques (964.80(160.63) vs. 860.77(234.07) mol/L, *p* = 0.001). However, none of these associations were retrieved for GlycB (*p* = 0.449, *p* = 0.180, *p* = 0.121, *p* = 0.112, and *p* = 0.080, respectively). These results confirm an association between GlycA levels and subclinical CV disease in RA. Furthermore, GlycA was observed to be independently associated with subclinical CV disease after controlling for traditional CV risk factors (age, sex, hypertension (HTA), dyslipidemia, smoking diabetes, and body mass index (BMI)) (Table 3). Moreover, this association remained after adjusting for CRP as well (Table S2).

**Figure 2:**
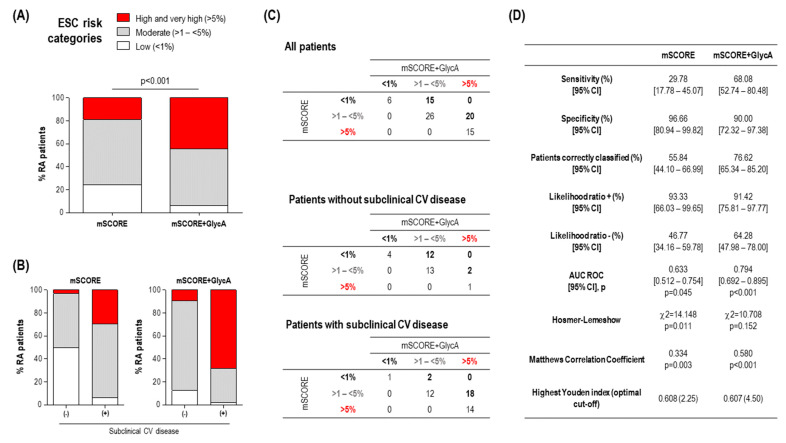
GlycA improved CV risk stratification in early RA. (**A**) Adding GlycA to the mSCORE increased the proportion of early RA patients reaching the high and very high risk categories. Differences between groups were assessed by 2 test. (**B**) Adding GlycA to the mSCORE was shown to reclassify a considerable proportion of patients into a more appropriate CV risk group according to their subclinical CV disease status, compared with mSCORE alone. (**C**) Comparative analyses of the stratification based on risk groups of the mSCORE and mSCORE + GlycA. Numbers in the tables indicate the number of individuals according to each mSCORE/mSCORE + GlycA status. Numbers below or above the diagonal correspond to reclassified patients (highlighted in bold). (**D**) Comparative classification and calibration metrics for mSCORE and mSCORE + GlycA for the identification of patients with subclinical CV disease. Analyses were made according to the statistics presented in the first column. AUC ROC, area under the receiver operating characteristic curve.

Next, whether GlycA levels could be of clinical relevance to identify patients with subclinical CV disease, alone or in combination with traditional CV risk factors, was assessed. GlycA alone was able to discriminate the presence of subclinical CV disease (AUC ROC (95% CI), *p*: 0.658(0.538–0.777), *p* = 0.018). Adding GlycA quartiles to the mSCORE improved the identification of subclinical CV disease (Figure 2). Only 18.9% of RA patients were classified as having high/very high risk based on the mSCORE (Figure 2A), whereas adding GlycA increased this figure to 44.3%. This change was more evident in patients with subclinical CV disease (29.7% vs. 68.9%), whereas it has a negligible effect in those without (3.12% vs. 9.37%) (Figure 2B). Adding GlycA to the mSCORE allowed to reclassify 35 patients to higher risk categories, whereas it did not result in reclassification to lower risk strata (Figure 2C). Interestingly, most of the patients reaching the high risk category exhibited plaque occurrence (18/20), whereas those reaching the moderate cut-off were mostly free of subclinical CV disease (2/15) (Figure 2C). As a consequence, adding GlycA to the mSCORE resulted in a better discrimination capacity (AUC ROC) (Figure 2D) and was found to provide additional value to the discrimination (difference between areas (De Long statistic): 0.172 (0.096–0.247), *p* < 0.0001). Moreover, adding GlycA to the mSCORE improved classification metrics (sensitivity, rate of patients correctly classified, and Matthews correlation coefficient) and also improved the risk prediction (Hosmer–Lemeshow statistic) (Figure 2D). More importantly, despite reaching similar highest Youden indices, the optimal cut-off value achieved by adding GlycA to mSCORE was more realistic for stratification than that of mSCORE alone (Figure 2D), which was mostly specificity-skewed. In order to further quantify the improvement resulting from adding GlycA to the mSCORE, the NRI and IDI were computed from the predicted risks retrieved in logistic regression models. Adding GlycA to the mSCORE yielded a NRI of 0.587 (0.419–0.753) (*p* = 0.011) with an IDI equal to 0.225 (0.137–0.312) (*p* < 0.001), hence confirming the GlycA-driven improvement in the classification.

On the basis of these findings, whether GlycA levels on their own were the only, direct responsible of this reclassification or if, on the contrary, they served as a surrogate marker of additional mediators beyond the traditional CV risk factors included in the mSCORE remains to be elucidated. In order to gain insight into the nature of the GlycA-driven reclassification, a subgroup analysis was performed. To this end, the 20 patients reclassified from mSCORE moderate to the high risk category after adding GlycA were compared to those staying in the original moderate risk category (*n* = 26) (Figure 2C). Interestingly, reclassified patients showed a similar cardiometabolic risk profile, except for being slightly older and more likely hypertensive (Table S3). Similarly, no differences in clinical features were noted. However, when the advanced lipoprotein profile was analysed, reclassified patients exhibited strong differences in VLDL-C and IDL-C levels, as well as higher triglyceride content in all the lipoprotein fractions. Additionally, increased levels of small LDL-P and a higher VLDL particle diameter was found (Table S3). Equivalent results were obtained if reclassified patients were compared to the whole group of low/moderate risk patients (*n* = 47). Furthermore, when the low to moderate reclassification was analysed, it was observed that reclassified patients exhibited slightly altered VLDL levels and systemic inflammation (Table S4), although reduced in absolute terms. Importantly, reclassified patients exhibited similar figures when compared with those initially classified as moderate risk by both scores (*n* = 26).

Taken together, these findings confirm that GlycA is related to atherosclerosis occurrence and vulnerability, and it could improve the CV risk stratification of early RA patients with high/very high risk over mSCORE alone by identifying a subgroup of patients with a more pro-atherogenic profile that cannot be captured by conventional approaches.

### 3.4. GlycA as a Biomarker of Treatment Outcome in Early RA Patients

Then, we studied whether GlycA levels could be a predictor of treatment response. Therefore, untreated early RA patients initiating csDMARD therapy (low-dose glucocorticoids and methotrexate in combination) were followed for 6 (*n* = 50) and 12 months (*n* = 42) and DAS28 remission rates were registered.

**Figure 3:**
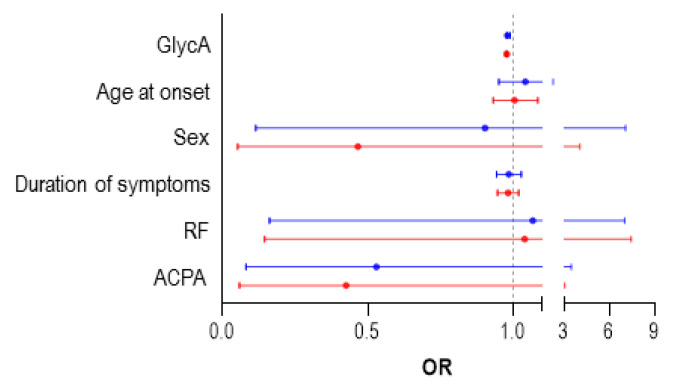
Predictors of Disease Activity Score 28-joints (DAS28) remission in treatment-naïve RA patients. Forest plot showing the odds ratio (OR) and 95% confidence interval (CI) of the different predictors of DAS28 remission achievement at 6 (red) and 12 (blue) months in fully adjusted, multivariate regression models (Table 3). ACPA, Anti-citrullinated protein antibodies; RF, rheumatoid factor.

Patients achieving DAS28 remission at 6 months (*n* = 27) exhibited lower GlycA levels compared with those who did not achieve remission (*n* = 23) (819.81 (199.27) vs. 981.68 (237.85) mol/L, *p* = 0.006). AUC ROC analyses confirmed that GlycA levels could discriminate between patients reaching DAS28 remission at 6 months and those who did not (0.75 (0.611–0.880), *p* = 0.003). Univariate analyses demonstrate that baseline GlycA levels predicted remission at 6 months (Table 4). This association remained statistically significant after adjusting for potential confounders (Table 4) (Figure 3). Moreover, when remission at 12 months was investigated, similar results were obtained in ROC (0.699 (0.540–0.859), *p* = 0.020) and regression analyses (Table 4) (Figure 3). These associations were maintained even after adjusting for CRP levels at baseline (Table S5), thus confirming the independent effect of GlycA. Equivalent results were obtained when the EULAR good response criteria were modelled. On the other hand, although GlycB was associated with remission rates in univariate analyses, this association disappeared in multivariate models (Table S6).

These results support a role for GlycA as an independent predictor of csDMARD treatment response in early RA patients.

### 3.5. GlycA Levels Are Associated with Serum Glycosyltransferase Activity

Finally, in order to gain insight into the origin of the increased glycoprotein signals in early RA, the serum glycosyltransferase activity was assessed.

**Figure 4:**
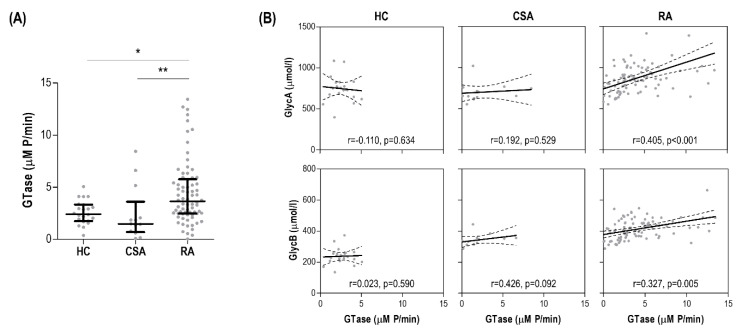
Glycoprotein signals and serum glycosyltransferase activity. (**A**) Glycosyltransfersase (GTase) activity was measured in serum samples and compared across groups. Differences were assessed by Kruskal–Wallis tests with Dunn–Bonferroni posthoc tests. The *p*-values from the latter were indicated as follows: * *p* < 0.050 and ** *p* < 0.010. (**B**) The associations between serum GTase (horizontal axis) and glycoprotein levels (vertical axis) in all the study groups were studied by correlation analyses (Spearman ranks’ tests) and indicated at the bottom of each graph.

Serum glycosyltransferase was significantly increased in early RA patients compared with CSA individuals and HC (Figure 4A). Interestingly, glycosyltransferase was not associated with traditional cardiometabolic risk factors (all *p* > 0.050), but a slight positive correlation with DAS28 was found (r = 0.249, *p* = 0.021). Moreover, glycosyltransferase activity was strongly correlated with GlycA serum levels, as well as those of GlycB to a lower degree, in early RA patients, whereas no associations were observed in the CSA or HC groups (Figure 4B).

**Figure 5:**
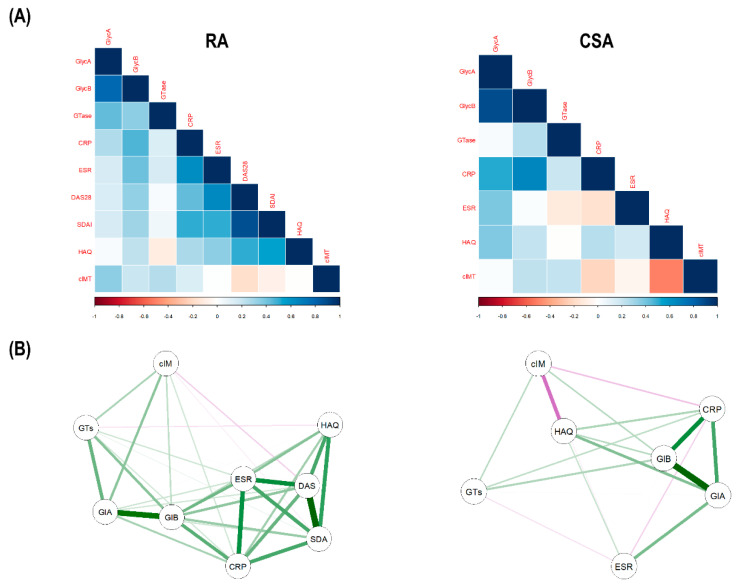
Integrative analyses of glycoprotein profiles, glycosyltransferase activity, and inflammatory markers. (**A**) The correlations among glycoprotein levels (GlycA and GlycB), glycosyltransfersase activity (GTase), inflammatory parameters (CRP and ESR), disease indices (DAS28, Simplified Disease Activity Index (SDAI), and Health Assessment Questionnaire (HAQ)) and carotid intima-media thickness (cIMT) were plotted in correlation matrices. In these correlograms, the colour of the tiles is proportional to the strength of the correlation between each pair of variables, according to the legend at the bottom. Names of the variables are indicated in red. (**B**) Network analyses depicted based on the correlations among variables. Each node corresponds to a variable (GlA: GlycA, GlB: GlycB, GTs: GTase, cIM: cIMT, DAS: DAS28, SDA: SDAI) and the lines between nodes illustrate the strength (width) and type (green: positive, red: negative) of the correlations between each pair of nodes. The relative position of the nodes parallels its degree of correlation, that is, nodes more closely correlated locate closer to each other.

Next, integrative analyses to visualize all these associations were performed. Correlation analyses confirmed strong correlations between GlycA and both GTase and cIMT in RA, but not in CSA. Moreover, GlycB was more strongly correlated with inflammatory markers and disease indices in RA (Figure 5A). Furthermore, network analyses confirmed this grouping pattern, where a clear compartmentalization was observed for the associations of GlycA and GlycB in RA (Figure 5B).

In conclusion, serum glycosyltransferase was observed to be increased in early RA patients and positively associated with GlycA and GlycB serum levels.

## 4. Discussion

Biomarkers enabling patient stratification and guiding the decision-making process in RA, especially for CV risk assessment, are a major unmet need. Current advances in high-throughput analytical approaches have allowed the identification of potential candidates to cover this need. In the present study, we report for the first time that GlycA levels are increased during the earliest phase of RA, but not in CSA, in parallel to serum glycosyltransferase activity. Taken together, the results reported herein point to a role of GlycA as a potential biomarker of CV risk and treatment response in early RA.

Our findings demonstrate a clear increase in glycoprotein signals, both GlycA and GlycB, during the early phase of RA. Previous (limited) evidence mostly came from cohorts with long-standing disease, with considerable rates of erosive disease and use of biologics, and without prospective follow up [7,10]. Therefore, our results expand this notion by confirming that elevated GlycA levels are an early event in RA course, thus ruling out an effect of disease duration or treatment exposure. This notion has key implications for its use as a biomarker, owing to the therapeutic relevance of the early stage. Importantly, GlycA levels were independently associated with therapy outcomes, even after adjusting by CRP levels and duration of symptoms, a major determinant of treatment response. Although the role of the ‘window of opportunity’ has been proposed, its exact delimitation is under debate [24]. It can be hypothesized that GlycA levels may assist in the identification of the window of opportunity, thus rethinking this concept from a pure calendar time to a more biological concept. Our results also allowed us to exclude the potential effect of confounders such as cardiometabolic risk factors or glucose homeostasis, as impaired glucose metabolism is a common hallmark of RA [25]. However, this had not been systematically addressed in this setting.

A remarkable result from our study was the comparative analysis between early RA and CSA. Raised GlycA levels were not observed in CSA, as opposed to early RA. This was in line with the serum levels of glycosyltransferase activity. Although previous studies reported altered glycosyltransferase activity in RA, the relevance of such findings was unexplored [26,27]. These findings may help to gain a better understanding of the role of inflammatory glycoproteins along RA development. Although some inflammatory and autoimmunity mediators are known to be increased prior to RA onset [28,29], enhanced glycoprotein emergence seems to be restricted to the clinical RA phase. Interestingly, increased liver production of glycosyltransferases has been documented to be triggered by acute inflammatory mediators [13]. This leads us to think that the increased pro-inflammatory milieu during the preclinical stage of RA could prompt an increase in glycosyltransferases that can result in increased glycoprotein levels, peaking during RA onset. Furthermore, increased protein glycosylation has been associated with functional protein changes, thus redirecting glycoproteins to different cellular and tissue receptors [30,31], hence causing aberrant responses. Moreover, glycosylation may also lead to changes in antigenicity [32,33], thus fuelling autoimmunity, which can in turn result in an increased production of several inflammatory mediators. Taken together, these lines of evidence may point to a positive feedback loop that can explain the connection between inflammation, glycosylation, and autoimmunity along the very early stages of RA. Actually, protein citrullination and the associated epitope spreading represent a similar roadmap that is now widely accepted for ACPA-positive RA [34]. Therefore, these notions suggest a role for protein glycosylation as a ‘second hit’ in the RA development. Whether inhibition of glycosyltransferases represents a potential therapeutic target to manage and/or prevent RA warrants further studies.

Of note, although GlycA and GlycB were associated with markers of systemic inflammation (CRP and ESR), the coefficient correlations were moderate (0.3–0.5), pointing only to a partial overlap. This finding suggests that, whereas both biomarkers may overlap in sensing similar aspects of systemic inflammation, they also cover different aspects of the inflammatory response [35,36]. Moreover, the former (especially GlycA) outperformed the latter in their role as biomarkers. This may be attributed to the integrative/aggregate nature of the GlycA NMR signal, which collates multiple proteins belonging to several pathways [6]. Consequently, GlycA may capture more information than a single-nature biomarker. Interestingly, the same rationale has already been proposed for the role of red cell distribution width as a biomarker compared with acute-phase reactants by our group [37] and others [38]. Moreover, acute-phase reactants, such as CRP, have several limitations that preclude their optimal use as biomarkers in chronic conditions [39]. Furthermore, CRP appears to increase with age [40], differences have also been observed for sex [41] and genetic determinants [42], and it is obviously strongly influenced by immunomodulatory treatments. As a result, some guidelines highlight the need for serial measurements rather than individual values to make therapeutic decisions [43,44]. However, current evidence demonstrate that GlycA can be considered a more robust, credible biomarker in different clinical contexts (reviewed in [13]). The findings reported herein could be a proof-of-concept study that supports the additional value of GlycA as a biomarker in early RA.

GlycA has received considerable attention in the area of CV risk assessment, although no studies have focused in RA. Our study demonstrated an association of GlycA levels not only with atherosclerosis occurrence, but also with plaque vulnerability. Although the link between inflammation and atherosclerosis is widely accepted, the exact underlying mediators are still to be identified. Increased glycosylation is associated with changes in endothelial cell functionality and, probably, vascular function [45]. Interestingly, complex molecular pathways linking IgG glycosylation to subclinical atherosclerosis have been reported [46]. All these lines of evidence may point to glycosylation as a potential missing link between inflammation and atherosclerosis from a mechanistic perspective.

CV risk stratification in current practice has been recognized to be suboptimal in RA and other rheumatic conditions. Although several efforts have been conducted to adapt risk scores in RA, the current management is far from being optimal, and the investigation of novel biomarkers for stratification is into the research agenda [15]. Attempts to develop disease-specific risk scores or include CRP levels in existing algorithms have failed to show a benefit in patient reclassification [47,48]. Our results demonstrate that adding GlycA to the mSCORE markedly improved the risk stratification and identification of patients with atherosclerosis. Then, GlycA may be of help to address the need of a biomarker to account for the effect of inflammation on CV risk. Interestingly, the reclassification guided by GlycA categories yielded an acceptable balance in sensitivity and specificity, with a considerably degree of accuracy. Interestingly, by adding GlycA, a correct classification of around 75% of the RA patients was reached, which is in line with the total CV risk that can be predicted in RA populations [49]. Moreover, the use of GlycA allowed the identification of a subgroup of patients with a profoundly impaired cholesterol metabolism, characterized by increased levels of triglyceride-rich lipoproteins that are highly atherogenic. This is supported by previous studies on congenital defects of glycosylation, which are associated with decreased LDL-C levels [50]. Moreover, altered lipoprotein functionality is a common hallmark of RA [51], and the precise identification of those with an atherogenic profile represents a crucial challenge in the clinical setting as it cannot be captured by conventional lipid analyses, thus emphasizing the usefulness of GlycA in this scenario.

Our results may be of interest beyond RA, as increased CV risk, altered cholesterol metabolism, and poor risk stratification are common hallmarks in the entire field of rheumatology and inflammatory conditions. As elevated GlycA levels have been described in other inflammatory conditions, adapting CV risk scores to include GlycA may be a broadly generalizable solution and will allow the use of an existing algorithm, instead of the use of disease-specific algorithms, which has been debated and adds complexity and pressure to the current clinical practice.

Overall, this study represents a step forward towards clinical applications of GlycA in the setting of existing algorithms. In addition to the advantages already commented on over CRP, GlycA quantification relies on a reliable method that avoids the intra- and inter-assay variability of (hs)CRP [52,53]. Additionally, it has been demonstrated to be more stable at room temperature and in frozen conditions. Although, in previous years, there was limited access to core facilities with H-NMR technology, this is becoming more available nowadays. Moreover, its longer stability and reliability may facilitate the logistic aspect behind their use as a biomarker (reviewed in [54]). Of note, GlycA testing may have important advantages over the use of ultrasound imaging techniques, as it can be automated and processed in batch, in a less time-consuming and operator bias-free way. Finally, the fact that NMR measurement allows a simultaneous lipoprotein characterization provides additional advantages, in relation to cost-effectiveness as well [6,54].

In summary, inflammatory glycoprotein signals are elevated during the earliest stage of RA, related to an altered glycosyltransferase activity, but not at the CSA stage. GlycA levels were found to be a potential biomarker to predict early response to csDMARD therapy and improved CV risk stratification in early RA patients, by re-classifying a group of patients with a highly atherogenic lipoprotein profile. Our study has relevant strengths, including a robust and detailed characterization of treatment-naïve early RA patients, follow up during the early phase (12 months), the inclusion of CSA individuals, and the advanced lipoprotein characterization.

This study comes with some limitations that must be remarked. The main limitation of this work is the use of subclinical CV disease as endpoint, instead of a hard CV endpoint. However, it must be noted that individuals with documented atherosclerosis have the same level of risk (high/very high) as patients with clinical CV disease [19]. Moreover, the best cut-offs for GlycA levels need to be investigated in larger studies. Additionally, whether GlycA levels may be a predictive biomarker for CV disease occurrence in the long-term cannot be evaluated with the current study design. Finally, whether the use of GlycA could be considered as cost-effective cannot be assessed in our study.

Therefore, our results must be interpreted as a proof-of-concept study about the clinical relevance of GlycA in this scenario, as some limitations exist. As such, it paves the ground for future studies to define the clinical perspectives and the added value of GlycA as a biomarker in early RA. On the one hand, because CV disease is a chronic, cumulative process, in order to validate the clinical relevance of GlycA as a biomarker, large-scale trials with a long-term follow up and an appropriate appraisal of time-adjusted confounders need to be performed to evaluate its role in predicting hard CV outcomes (such as myocardial infarction, stroke, angina requiring hospitalization, peripheral vascular disease, or CV death), by comparing GlycA-reclassification to standard care in managing CV risk. Moreover, as a robust algorithm to identify at onset patients with poor prognosis is lacking, it may be challenging to design a trial to evaluate the added value of GlycA in this setting. Owing to the long follow up required, a registry-based cohort of early patients looking at the rates of early remission upon csDMARD, usage of biologic drugs in the medium term, and occurrence of joint outcomes (including erosion) and comorbidities in the long-term is warranted. Then, trials evaluating whether GlycA measurement (and treatment-decision making to more severe drug approaches) compared with standard treatment algorithms would provide better figures of sustained disease control should be conceived. Furthermore, cost-effectiveness evaluations will be required in both indications (CV outcomes and disease management) in order to confirm the clinical relevance of GlycA.

## 5. Conclusions

GlycA levels were increased in early RA but not individuals with arthralgia, independently of traditional risk factors, and related to serum glycosyltransferase activity. GlycA was associated with subclinical atherosclerosis occurrence and plaque risk in early RA, and adding GlycA to the mSCORE improved the identification of patients at risk and helped to reclassify individuals to more appropriate risk categories. Impaired levels of lipoproteins that cannot be captured by conventional methods underlie this reclassification. GlycA also identified patients less likely to promptly respond to conventional csDMARD treatment. Thus, adding GlycA levels to the conventional algorithms helped to close the gap between the predicted risk and the actual risk, by capturing the influence of inflammation and impaired cholesterol metabolism. GlycA may be considered an emergent biomarker for disease stratification and decision-making in early RA.

## Figures and Tables

**Table 1:** Demographic, clinical, and subclinical atherosclerosis assessments in rheumatoid arthritis (RA) and clinically-suspect arthralgia (CSA).

Variables	CSA *n* = 14	RA *n* = 82
Age (years), mean ± SD	49.28 ± 10.53	58.51 ± 10.49
Sex (women/men)	14/0	66/16
***Clinical features***		
Duration of symptoms (weeks)	24.00 (40.00)	20.00 (22.00)
Morning stiffness (minutes)	30.00 (50.00)	60.00 (80.0)
Tender joint count	3.00 (3.00)	8.00 (7.00)
Swollen joint count	0.00 (1.00)	6.00 (5.00)
ESR (mm/h)	7.50 (8.84)	24.00 (27.00)
CRP (mg/dL)	0.15 (0.30)	0.80 (2.20)
Patient global assessment (VAS 0–100)	30.00 (50.00)	70.00 (25.00)
Pain assessment (VAS 0–10)	5.00 (5.00)	7.00 (2.00)
DAS28		5.40 (1.78)
SDAI		24.22 (16.90)
HAQ	0.55 (0.60)	1.11 (1.00)
Fatigue (VAS 0–10)	4.50 (5.00)	5.00 (7.00)
RF+, *n* (%)	8 (66.6)	57 (69.5)
ACPA+, *n* (%)	7 (58.3)	56 (68.2)
***Traditional CV risk factors***		
Hypertension, *n* (%)	1 (12.5)	28 (34.11)
Diabetes, *n* (%)	0 (0.0)	9 (10.9)
Dyslipidemia, *n* (%)	3 (37.5)	24 (29.2)
Smoking, *n* (%)	10 (71.4)	31 (27.8)
Obesity, *n* (%)	3 (37.5)	33 (40.2)
Waist circumference	92.00 (16.00)	101.00 (20.00)
***Glucose homeostasis features***		
Glucose (mg/dL)	98.00 (19.00)	92.00 (10.00)
Insulin (U/mL)	7.87 (4.57)	10.10 (11.80)
C-peptide (ng/mL)	2.10 (1.4)	2.79 (1.6)
HOMA-IR	1.00 (0.60)	1.30 (1.38)
QUICKI	0.36 (0.02)	0.33 (0.05)
***Subclinical atherosclerosis* (*n* = 92) **	n = 13	n = 79
cIMT (mm)	0.58 ± 0.15	0.67 ± 0.10
Plaque presence, *n* (%)	4 (30.7)	46 (58.2)
Plaque number	0.46 ± 0.87	0.96 ± 1.01
Plaque presence or cIMT > 0.90, *n* (%)	4 (30.7)	47 (59.6)
High-risk plaque	0 (0.0)	20 (25.3)
***Treatments, n (%)***		
None	14 (100)	69 (84.1)
Glucocorticoids	0 (0)	13 (15.8)
Methotrexate	0 (0)	5 (6.0)

Demographic, clinical features, traditional cardiovascular (CV) risk factors, and subclinical atherosclerosis measurements of RA patients and CSA subjects are summarized. Variables are expressed as median (interquartile range), mean ± SD, or *n* (%), according to the distribution of the variables. ACPA, Anti-citrullinated protein antibodies; DAS28, Disease Activity Score 28-joints (DAS28); SDAI, Simplified Disease Activity Index; HAQ, Health Assessment Questionnaire; cIMT, carotid intima-media thickness; RF, rheumatoid factor; CPR, C-reactive protein; ESR, erythrocyte sedimentation rate; HOMA-IR, Homeostatic Model Assessment of Insulin Resistance; QUICKI, the Quantitative Insulin Sensitivity Check Index.

**Table 2:** Glycoproteins and cardiometabolic factors in early RA patients.

Variables	GlycA	GlycB
***Traditional CV Risk Factors***		
Hypertension	*p* = 0.115	*p* = 0.374
Diabetes	*p* = 0.078	*p* = 0.090
Dyslipidemia	*p* = 0.163	*p* = 0.536
Smoking	*p* = 0.977	*p* = 0.793
Obesity	*p* = 0.107	*p* = 0.113
Waist circumference	r = 0.145 *p* = 0.233	r = 0.078 *p* = 0.524
***Glucose Homeostasis Parameters***		
Glucose	r = 0.068 *p* = 0.542	r = 0.218 *p* = 0.050
Insulin	r = 0.087 *p* = 0.485	r = −0.001 *p* = 0.994
C-peptide	r = −0.034 *p* = 0.786	r = −0.075 *p* = 0.545
HOMA-IR	r = 0.068 *p* = 0.593	r = 0.002 *p* = 0.990
QUICKI	r = −0.181 *p* = 0.147	r = −0.135 *p* = 0.279

Analyses of the correlations between GlycA and GlycB with traditional CV risk factors and glucose homeostasis parameters. Differences were assessed by Mann–Withney U tests, whereas correlations were analyzed by Spearman’s rank tests. These findings confirm that increased GlycA levels cannot be attributed to impaired glucose metabolism or cardiometabolic risk factors in early RA.

**Table 3:** GlycA as an independent predictor of subclinical CV disease in early RA.

Models	OR	95% CI	*p*-Value
***Univariate***			
GlycA, per unit	1.004	1.002–1.007	0.020
***Multivariate***			
GlycA, per unit	1.008	1.003–1.012	0.023
Age at sampling, per year	1.091	1.017–1.169	0.015
Sex, women	4.609	0.635–33.427	0.131
Hypertension, yes	3.811	0.752–19.305	0.106
Smoking, yes	2.491	0.908–6.833	0.076
Dyslipidemia, yes	1.093	0.250–4.776	0.906
Diabetes, yes	3.731	0.345–40.371	0.278
BMI, per unit	0.013	0.012–22.705	0.254

The association between GlycA and the presence of subclinical CV disease was analysed by univariate and multivariate logistic regression analyses. Multivariate models were fully adjusted for all the potential confounders listed in the table. CI, confidence interval; OR, odds ratio; BMI, body mass index.

**Table 4:** GlycA as predictor of treatment outcomes in early RA.

Model	6 Months			12 Months		
	OR	95% CI	*p*-Value	OR	95% CI	*p*-Value
***Univariate***						
GlycA, per unit	0.993	0.988–0.998	0.008	0.994	0.989–0.999	0.015
***Multivariate***						
GlycA, per unit	0.992	0.986–0.998	0.015	0.993	0.986–0.999	0.030
Age at onset, per year	1.005	0.931–1.085	0.894	1.042	0.951–1.142	0.381
Sex, women	0.466	0.053–4.055	0.489	0.903	0.115–7.079	0.903
Duration of symptoms, per week	0.982	0.946–1.019	0.342	0.985	0.943–1.028	0.489
RF, +	0.426	0.059–3.055	0.396	0.530	0.081–3.482	0.509
ACPA, +	1.039	0.146–7.417	0.696	1.067	0.162–7.024	0.946

The role of GlycA as predictor of treatment outcomes upon conventional synthetic disease-modifying antirheumatic drug (csDMARD) treatment in treatment-naïve, early RA patients was analysed by univariate and multivariate logistic regression analyses. Multivariate models were fully adjusted for all the confounders listed in the table. DAS28 remission status at 6 and 12 months after treatment initiation was entered as the dependent variable.

## Supplementary Materials

The following are available online at https://www.mdpi.com/2077-0383/9/8/2472/s1, Table S1: Lipoprotein characterization by 1H-NMR; Table S2: Analysis of the GlycA-attributed moderate to high reclassification; Table S3: Analysis of the GlycA-attributed low to moderate reclassification; Table S4: GlycB as predictor of treatment outcomes in early RA.
