# Supplementary material for: GlycA Levels during the Earliest Stages of Rheumatoid Arthritis: Potential Use as a Biomarker of Subclinical Cardiovascular Disease
Source: J Clin Med. 2020 Aug 1;9(8):2472. doi: 10.3390/jcm9082472 (PMC7463667; doi:10.3390/jcm9082472)
Supplement: Supplementary file 1 [file jcm-09-02472-s001.pdf]

Table S1. Lipoprotein characterization by ^{1}H-NMR. An advanced characterization of the lipoprotein profile was performed by H-NMR in all study subjects. Differences were assessed by ANOVA test. Variables were summarized as mean ± SD.

	**HC** *n* = 28	**CSA** *n* = 14	**RA** *n* = 82	***p*-Value**
Lipoprotein profile				
VLDL-C (mg/dl)	12.93±7.86	13.07±7.64	17.50±12.78	0.116
IDL-C (mg/dl)	9.66±4.08	9.43±4.42	11.74±5.13	0.076
LDL-C (mg/dl)	133.49±23.65	133.51±26.67	136.62±30.79	0.851
HDL-C (mg/dl)	57.53±11.91	58.70±11.90	53.69±12.53	0.186
VLDL-TG (mg/dl)	62.46±28.97	60.90±32.89	78.94±63.97	0.270
IDL-TG (mg/dl)	10.58±3.36	10.04±3.49	12.07±3.77	0.055
LDL-TG (mg/dl)	15.51±4.63	15.67±4.34	17.79±5.44	0.079
HDL-TG (mg/dl)	16.03±4.14	15.28±5.99	16.63±4.81	0.577
VLDL-P (nmol/l)	45.90±22.90	44.20±24.48	57.90±43.04	0.216
Large VLDL-P (nmol/l)	1.21±0.50	1.14±0.51	1.47±0.80	0.141
Medium VLDL-P (nmol/l)	4.08±2.28	4.66±2.34	5.79±7.21	0.405
Small VLDL-P (nmol/l)	40.62±21.09	38.36±21.84	50.63±36.11	0.207
LDL-P (nmol/l)	1351.98±268.29	1339.99±283.15	1400.82±321.86	0.658
Large LDL-P (nmol/l)	186.78±26.28	188.17±33.89	189.20±37.76	0.951
Medium LDL-P (nmol/l)	394.18±98.42	408.57±111.69	422.90±140.32	0.588
Small LDL-P (nmol/l)	771.15±178.04	743.23±193.69	788.71±206.98	0.710
HDL-P (μmol/l)	29.27±4.60	29.51±4.36	27.78±5.25	0.258
Large HDL-P (μmol/l)	0.26±0.04	0.26±0.03	0.27±0.04	0.685
Medium HDL-P (μmol/l)	9.79±1.81	9.97±1.52	9.54±1.59	0.581
Small HDL-P (μmol/l)	19.18±3.31	19.27±3.33	9.54±1.59	0.262
VLDL diameter (nm)	42.00±0.56	42.18±0.34	42.03±0.42	0.466
LDL diameter (nm)	21.02±0.28	21.11±0.35	21.03±0.38	0.758
HDL diameter (nm)	8.26±0.09	8.27±0.06	8.29±0.10	0.266

Table S2. Analysis of the GlycA-attributed moderate to high reclassification. Demographic parameters, cardiometabolic factors, clinical characteristics and advanced lipoprotein features were compared between patients reclassified to the high risk category (*n* = 20) and those staying in the moderate category (*n* = 26) after adding GlycA to the mSCORE. Differences were assessed by Mann-Withney U tests, χ2 tests or t tests, as appropriate. Variables were summarized as median (interquartile range), mean ± SD or *n*(%), as appropriate.

	**High Risk-reclassified (*n* = 20)**	**Moderate Risk Non-reclassified (*n* = 26)**	***p*-Value**
Age (years), mean (range)	64.29 (49.75-83.67)	57.79 (48.08-76.50)	0.058
Gender (female/male)	17/3	22/4	0.971
Clinical features			
Duration of symptoms (weeks)	14.00 (14.00)	24.00 (22.00)	0.436
Morning stiffness (minutes)	60.00 (97.50)	45.00 (83.80)	0.551
Tender Joint Count	8.50 (9.00)	10.00 (8.00)	0.955
Swollen Joint Count	6.00 (6.00)	5.50 (6.00)	0.547
ESR (mm/h)	25.50 (20.00)	24.50 (27.00)	0.557
CRP (mg/dl)	1.35 (3.50)	0.90 (2.00)	0.106
Patient Global Assessment (VAS 0-100)	70.00 (38.00)	80.00 (20.00)	0.362
Pain Assessment (VAS 0-10)	7.00 (3.00)	7.00 (2.00)	0.479
DAS28	5.42 (1.60)	5.57 (1.04)	0.727
SDAI	28.15 (16.00)	30.30 (16.30)	0.850
HAQ	1.60 (1.00)	1.10 (1.00)	0.672
Fatigue (VAS 0-10)	5.00 (7.00)	6.00 (8.00)	0.532
RF+, n(%)	12 (60.0)	20 (76.9)	0.216
ACPA+, n(%)	12 (60.0)	17 (65.3)	0.577
Traditional CV risk factors			
Hypertension, n(%)	12 (60.0)	6 (23.0)	0.016
Diabetes, n(%)	3 (15.0)	2 (7.69)	0.430
Dyslipidemia, n(%)	8 (40.0)	8 (30.7)	0.450
Smoking, n(%)	7 (35.0)	9 (34.6)	0.878
Obesity, n(%)	11 (55.0)	11 (42.3)	0.432
Waist circumference	105.50 (22.00)	100.00 (23.50)	0.333
Glucose homeostasis features			
Glucose (mg/dl)	100.00 (22.00)	99.00 (13.00)	0.764
Insulin (μU/ml)	11.45 (16.20)	9.90 (12.10)	0.151
C-peptide (ng/ml)	3.20 (1.50)	2.61 (2.10)	0.660
HOMA-IR	1.50 (2.03)	1.30 (1.65)	0.175
QUICKI	0.32 (0.04)	0.33 (0.06)	0.112
Lipoprotein profile			
VLDL-C (mg/dl)	20.31±9.61	12.24±7.97	0.003
IDL-C (mg/dl)	13.75±5.05	10.36±4.13	0.016
LDL-C (mg/dl)	148.28±27.39	138.85±30.91	0.288
HDL-C (mg/dl)	56.13±14.00	57.30±12.70	0.768
VLDL-TG (mg/dl)	85.67±38.42	58.47±30.00	0.010
IDL-TG (mg/dl)	13.92±4.06	10.93±2.99	0.006
LDL-TG (mg/dl)	20.63±5.96	17.43±4.55	0..045
HDL-TG (mg/dl)	18.79±5.60	16.01±4.18	0.060
VLDL-P (nmol/l)	64.50±28.29	42.72±23.86	0.007
Large VLDL-P (nmol/l)	1.55±0.58	1.15±0.51	0.018
Medium VLDL-P (nmol/l)	5.96±3.19	4.08±1.91	0.017
Small VLDL-P (nmol/l)	56.99±27.49	37.48±21.95	0.007
LDL-P (nmol/l)	1540.67±296.25	1396.31±308.28	0.117
Large LDL-P (nmol/l)	201.18±32.03	195.00±35.43	0.544
Medium LDL-P (nmol/l)	471.80±116.17	445.49±142.48	0.506
Small LDL-P (nmol/l)	867.68±217.05	745.82±176.00	0.050
HDL-P (μmol/l)	29.39±5.79	28.72±5.15	0.684
Large HDL-P (μmol/l)	0.29±0.05	0.27±0.05	0.193

Medium HDL-P (μmol/l)	9.76±1.90	10.15±1.66	0.466
Small HDL-P (μmol/l)	19.32±4.37	18.29±4.21	0.423
VLDL diameter (nm)	41.86±0.20	42.14±0.58	0.028
LDL diameter (nm)	21.00±0.35	21.17±0.36	0.134
HDL diameter (nm)	8.27±0.08	8.32±0.12	0.110

Table S3. Analysis of the GlycA-attributed low to moderate reclassification. Demographic parameters, cardiometabolic factors, clinical characteristics and advanced lipoprotein features were compared between patients reclassified to the moderate risk category (*n* = 15) and those staying in the low category (*n* = 6) after adding GlycA to the mSCORE. Differences were assessed by Mann-Withney U tests, χ2 tests, Fischer exact tests, or t tests, as appropriate. Variables were summarized as median (interquartile range), mean ± SD or *n*(%), as appropriate.

	**Moderate risk-reclassified (*n* = 15)**	**Low risk non-reclassified (*n* = 6)**	***p*-Value**
Age (years), mean (range)	44.25 (30.33 – 71.42)	44.83 (37.33-52.33)	0.910
Gender (female/male)	14/1	6/0	1.000
Clinical features			
Duration of symptoms (weeks)	17.00 (21.00)	19.00 (16.00)	0.132
Morning stiffness (minutes)	60.00 (95.00)	62.50 (50.00)	0.302
Tender Joint Count	8.50 (5.00)	5.50 (3.00)	0.036
Swollen Joint Count	5.50 (5.00)	4.50 (3.00)	0.302
ESR (mm/h)	35.00 (38.00)	17.50 (26.00)	0.005
CRP (mg/dl)	0.90 (1.10)	0.15 (1.00)	0.008
Patient Global Assessment (VAS 0-100)	75.00 (33.00)	50.00 (35.00)	0.003
Pain Assessment (VAS 0-10)	7.50 (2.00)	4.50 (2.00)	0.011
DAS28	5.57 (1.63)	4.46 (1.12)	0.001
SDAI	27.60 (9.50)	11.10 (6.00)	0.002
HAQ	1.40 (1.00)	0.35 (1.00)	0.002
Fatigue (VAS 0-10)	7.00 (3.00)	3.00 (4.00)	0.645
RF+, n(%)	12 (80.0)	3 (50.0)	0.291
ACPA+, n(%)	12 (80.0)	4 (66.6)	0.598
Traditional CV risk factors			
Hypertension, n(%)	2 (13.3)	0 (0.0)	0.347
Diabetes, n(%)	0 (0.0)	0 (0.0)	-
Dyslipidemia, n(%)	2 (13.3)	0 (0.0)	0.347
Smoking, n(%)	8 (53.3)	0 (0.0)	0.046
Obesity, n(%)	5 (30.0)	2 (33.3)	1.000
Waist circumference	102.00 (16.25)	82.00 (20.00)	0.333
Glucose homeostasis features			
Glucose (mg/dl)	91.00 (19.00)	89.00 (15.00)	0.970
Insulin (μU/ml)	10.15 (8.10)	6.65 (5.00)	0.549
C-peptide (ng/ml)	2.61 (1.70)	1.97 (1.25)	0.442
HOMA-IR	1.30 (1.13)	0.85 (1.00)	0.549
QUICKI	0.33 (0.06)	0.36 (0.04)	0.350
Lipoprotein profile			
VLDL-C (mg/dl)	15.72±8.83	8.87±3.84	0.023
IDL-C (mg/dl)	10.32±4.03	8.12±2.83	0.239
LDL-C (mg/dl)	134.91±22.66	129.37±29.61	0.648
HDL-C (mg/dl)	51.86±11.34	53.78±8.65	0.714
VLDL-TG (mg/dl)	65.94±29.67	41.91±14.27	0.076
IDL-TG (mg/dl)	11.18±3.30	8.51±1.97	0.083
LDL-TG (mg/dl)	16.28±3.98	14.37±4.72	0.358
HDL-TG (mg/dl)	15.91±4.33	14.85±3.85	0.608
VLDL-P (nmol/l)	49.50±24.19	30.39±10.68	0.081
Large VLDL-P (nmol/l)	1.35±0.44	0.91±0.40	0.052
Medium VLDL-P (nmol/l)	4.50±1.78	3.10±1.26	0.099
Small VLDL-P (nmol/l)	43.65±22.15	26.37±9.78	0.023
LDL-P (nmol/l)	1367.93±232.10	1266.55±264.75	0.395
Large LDL-P (nmol/l)	188.31±30.78	192.20±37.93	0.809
Medium LDL-P (nmol/l)	409.75±96.39	394.74±170.03	0.799
Small LDL-P (nmol/l)	769.86±135.77	679.60±69.31	0.142
HDL-P (μmol/l)	27.28±5.21	26.34±4.19	0.699
Large HDL-P (μmol/l)	0.25±0.03	0.27±0.03	0.174

Medium HDL-P (μmol/l)	9.01±1.01	10.30±0.55	0.010
Small HDL-P (μmol/l)	18.01±4.51	15.76±3.91	0.299
VLDL diameter (nm)	42.02±0.29	42.15±0.43	0.423
LDL diameter (nm)	21.03±0.24	21.18±0.28	0.241
HDL diameter (nm)	8.27±0.07	8.37±0.10	0.059

Table S4. GlycB as predictor of treatment outcomes in early RA. The role of GlycB as predictor of treatment outcomes upon csDMARD treatment in treatment-naïve, early RA patients was analysed by univariate and multivariate logistic regression analyses. DAS28 remission status at 6 and 12 months after treatment initiation was entered as the dependent variable.

	**6 months**			**12 months**		
	**OR**	**95% CI**	***p*-value**	**OR**	**95% CI**	***p*-value**
Univariate						
GlycB, per unit	0.988	0.979 – 0.998	0.015	0.990	0.981 – 0.999	0.048
Multivariate						
GlycB, per unit	0.989	0.979 – 1.001	0.051	0.900	0.980 – 1.002	0.058
Age at onset, per year	1.002	0.932 – 1.076	0.966	1.030	0.949 – 1.117	0.479
Gender, female	0.781	0.131 – 4.659	0.786	1.505	0.241 – 9.348	0.662
Duration of symptoms, per week	0.988	0.950 – 1.020	0.388	0.987	0.947 – 1.028	0.520
RF, +	0.465	0.080 – 2.702	0.465	0.528	0.092 – 3.027	0.473
ACPA, +	1.464	0.252 – 8.511	0.671	1.535	0.260 – 9.065	0.636
